# An Antimicrobial Compound Isolated from *Cinnamomum Iners* Leaves with Activity against Methicillin-Resistant *Staphylococcus Aureus*

**DOI:** 10.3390/molecules16043037

**Published:** 2011-04-08

**Authors:** Fazlina Mustaffa, Jayant Indurkar, Sabariah Ismail, Marina Shah, Sharif Mahsufi Mansor

**Affiliations:** Centre for Drug Research, Universiti Sains Malaysia, 11800 Penang, Malaysia

**Keywords:** *Cinnamomum iners*, antimicrobial activity, xanthorrhizol

## Abstract

This study was designed to investigate the antimicrobial activity of *Cinnamomum iners* standardized leave methanolic extract (CSLE), its fractions and isolated compounds. CSLE and fractions were subjected to disc diffusion, minimum inhibitory concentration (MIC) and minimum bactericidal concentration (MBC) tests using different Gram positive and Gram negative bacteria and yeast. Within the series of fractions tested, the ethyl acetate fraction was the most active, particularly against methicillin resistant *Staphylococcus aureus* (MRSA) and *Escherichia coli*, with MIC values of 100 and 200 µg/mL, respectively. The active compound in this fraction was isolated and identified as xanthorrhizol [5-(1, 5-dimethyl-4-hexenyl)-2-methylphenol] by various spectroscopic techniques. The overall results of this study provide evidence that *Cinnamomum iners* leaves extract as well as the isolated compound xanthorrhizol exhibit antimicrobial activity for both Gram negative and Gram positive pathogens, especially against MRSA strains.

## 1. Introduction

The development of bacterial strain resistance against several antibiotics due to their uncontrolled usage has raised concern and interest in screening local medicinal plants as an alternative source of potential antimicrobial agents [1,2,3,4]. Methicillin resistant *Staphylococcus aureus* (MRSA) and *Escherichia coli* are the most common pathogens encountered in clinical specimens from inpatients and outpatients [5]. MRSA is also an opportunistic pathogen prominent in both hospital and community settings [6]. Hence, these studies are very important in discovering effective but at low cost antimicrobial compounds, especially against MRSA.

*Cinnamomum iners* is an evergreen tree belonging to the family Lauraceae. According to the Agroforestry Tree Database, *C. iners* is commonly found in India, Myanmar, Thailand, Malaysia, Indonesia and Southern Philippines. The major bioactive compounds of this plant are saponins, terpenes, cinnamic aldehyde and eugenol [7]. Ethnobotanical reports offer information on the medicinal properties of *C. iners* that include its antiplasmodial, cytotoxicity, amylase inhibitor, antinociceptive and anti-inflammatory activity [8,9,10]. Recently, our research team has reported the analgesic, antioxidant and toxicity of this plant’s leaves [11,12]. This plant is widely used as a traditional medicine to relieve headaches, breathing and appetite problems. This plant has also been used over the centuries on several illnesses with bacterial symptoms such as fevers, digestive ailments and coughs [13]. This suggests that this plant might contain bioactive compounds that act as antimicrobial agents. Therefore, the aim of this study was to isolate possible antimicrobial compounds from *C. iners* leaves.

## 2. Results and Discussion

### 2.1. Antimicrobial Activity

The antimicrobial activity results for *Cinnamomum iners* standardized leaves methanolic extract (CSLE), fractions and standard antibiotics against several pathogens are listed in Table 1. CSLE exhibited antimicrobial effects towards all the tested bacteria and yeast in the disc diffusion test. However, various fractions showed inhibitory activity on specific tested microorganisms. This may be due to synergism of the compounds present in CSLE. The ethyl acetate CSLE fraction showed potential antibacterial activity towards all the tested Gram positive and Gram negative bacteria, but it failed to inhibit the growth of tested yeast. Both the hexane and aqueous fractions inhibit Gram negative *P. aeruginosa*. The aqueous fraction also shows inhibition on *B. subtilis* and *S. sonnei*. The butanol fraction was capable of inhibiting a number of bacteria and yeast such as *B. cereus*, *P. aeruginosa*, *E.coli*, *S. sonnei*, *C. albicans* and *S. cerevisiae*. The standard antibiotic chloramphenicol inhibited all the tested bacteria and miconazole nitrate inhibited the tested yeast.

The antimicrobial activity of CSLE and its various fractions were quantitatively assessed by determining their MIC and MBC, as illustrated in Table 2. The MIC and MBC values of CSLE varied in the range of 0.78-50.00 mg/mL. The lowest MIC of CSLE (0.78 mg/mL) was recorded against MRSA and *E. coli*. In the case of the different solvent fractions, the ethyl acetate one showed significant antimicrobial activity against MRSA and *E. coli*, with MIC values of 100 and 200 µg/mL, respectively. Neither the hexane nor the aqueous fraction possessed effective inhibitory activity against any of the tested microorganisms, with MIC values more than 800 µg/mL. The butanol fraction showed excellent activity towards *E. coli*, compared to other CSLE fractions, MIC and MBC values of 100 µg/mL and 400 µg/mL, respectively, but it showed only moderate antibacterial activity against *B. cereus*, with MIC and MBC values of 400 and >800 µg/mL.

**Table 1:** Antimicrobial activity of CSLE, fractions and standard antibiotics by the disc diffusion method.

M/o	CSLE	Fractions				Standards		Solvent control (100% methanol)
		Ethyl acetate	Hexane	Aqueous	Butanol	Chloramphenicol	Miconazole nitrate	
Gram positive bacteria								
1	+	+	-	-	+	+	-	-
2	+	+	-	+	-	+	-	-
3	+	+	-	-	-	+	-	-
4	+	+	-	-	-	+	-	-
Gram negative bacteria								
5	+	+	-	-	+	+	-	-
6	+	+	+	+	+	+	-	-
7	+	+	-	+	+	+	-	-
Yeast								-
8	+	-	-	-	+	-	+	-
9	+	-	-	-	+	-	+	-

M/o: Microorganisms. (+): Susceptibility (inhibition zone ≥7 mm). (-): Absence of susceptibility. CSLE: *Cinnamomum iners* standardized leaves methanolic extract. **1**: *Bacillus cereus*. **2**: *Bacillus subtilis*. **3**: *Salmonella typhi*. **4**: methicillin resistant *Staphylococcus aureus* (MRSA). **5**: *Escherichia coli.* **6**: *Pseudomonas aeruginosa*. **7**: *Shigella sonnei*. **8**: *Candida albicans*. **9**: *Saccharomyces cerevisiae*.

**Table 2:** Minimum inhibitory concentration (MIC) and Minimum bactericidal concentration (MBC) of CSLE, fractions and standard antibiotics against microorganisms.

M/o		Fractions (µg/mL)				Standards (µg/mL)		
	CSLE (mg/mL)	Ethyl acetate	Hexane	Aqueous	Butanol	Chloramphenicol		Miconazole nitrate
	MIC	MIC	MIC	MIC	MIC	MIC	MBC	MIC
	MBC	MBC	MBC	MBC	MBC			MBC
Gram positive bacteria								
1	12.50	>800	>800	>800	400	6.25	12.5	NT
	25.00	>800	>800	>800	>800			NT
2	3.13	>800	>800	>800	>800	6.25	12.5	NT
	6.25	>800	>800	>800	>800			NT
3	6.25	>800	>800	>800	>800	6.25	12.5	NT
	12.50	>800	>800	>800	>800			NT
4	0.78	100	>800	>800	>800	6.25	12.5	NT
	1.50	200	>800	>800	>800			NT
Gram negative bacteria								
5	0.78	200	>800	>800	100	6.25	12.5	NT
	1.50	400	>800	>800	400			NT
6	25.00	>800	>800	>800	>800	6.25	12.5	NT
	50.00	>800	>800	>800	>800			NT
7	6.25	>800	>800	>800	>800	6.25	12.5	NT
	12.50	>800	>800	>800	>800			NT
Yeast								
8	3.13	>800	>800	>800	>800	NT	NT	6.25
	6.25	>800	>800	>800	>800			12.5
9	25.00	>800	>800	>800	>800	NT	NT	6.25
	50.00	>800	>800	>800	>800			12.5

M/o: Microorganism. CSLE: *Cinnamomum iners* standardized leaves methanolic extract. NT: Not tested. MIC: Minimum inhibitory concentration. MBC: Minimum bactericidal concentration. **1**: *Bacillus cereus*. **2**: *Bacillus subtilis*. **3**: *Salmonella typhi.* **4**: methicillin resistant *Staphylococcus aureus* (MRSA)*.* **5**: *Escherichia coli.* **6**: *Pseudomonas aeruginosa*. **7**: *Shigella sonnei*. **8**: *Candida albicans*. **9**: *Saccharomyces cerevisiae.*

The bioautography technique has been used to identify the bioactive constituents present in various fractions of the CSLE. Inhibition zones of antibacterial components were observed as white spots on a purple red background. White areas indicate the presence of antibacterial components that inhibited the growth of bacteria that did not support the reduction of INT to the coloured formazan [14].

**Figure 1:**
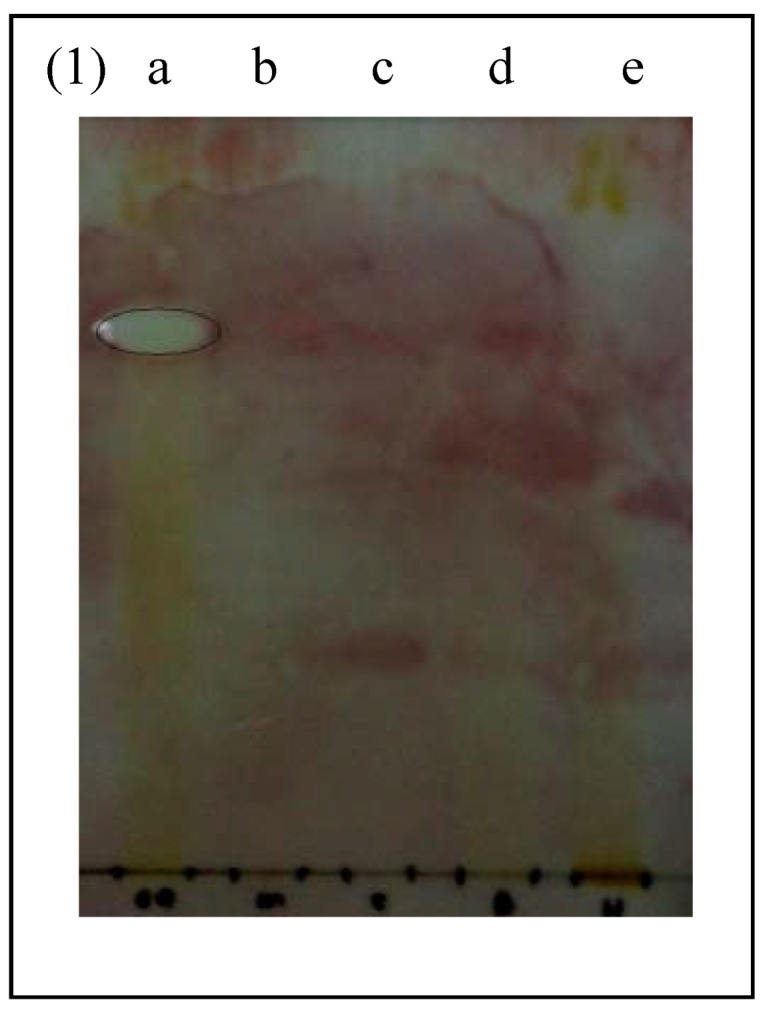
Bioautogram of fractions and antibiotics sprayed with actively growing methicillin resistant *Staphylococcus aureus* (MRSA). White areas indicate zones of growth inhibition. **a**: ethyl acetate fraction. **b**: methicillin. **c**: vancomycin. **d**: butanol fraction. **e**: hexane fraction.

The microbial strains for bioautography were selected based on their sensitivity to CSLE and fractions. The CSLE and ethyl acetate fractions demonstrated the lowest MIC against MRSA, whereas the CSLE and butanol fractions exhibited promising MIC towards *E. coli*. Hence, both of these bacteria (MRSA and *E. coli*) were selected for the bioautography assay. The bioautography assay exhibited inhibition zones (Rf value = 0.77) for ethyl acetate fraction against MRSA (Figure 1).

**Figure 2:**
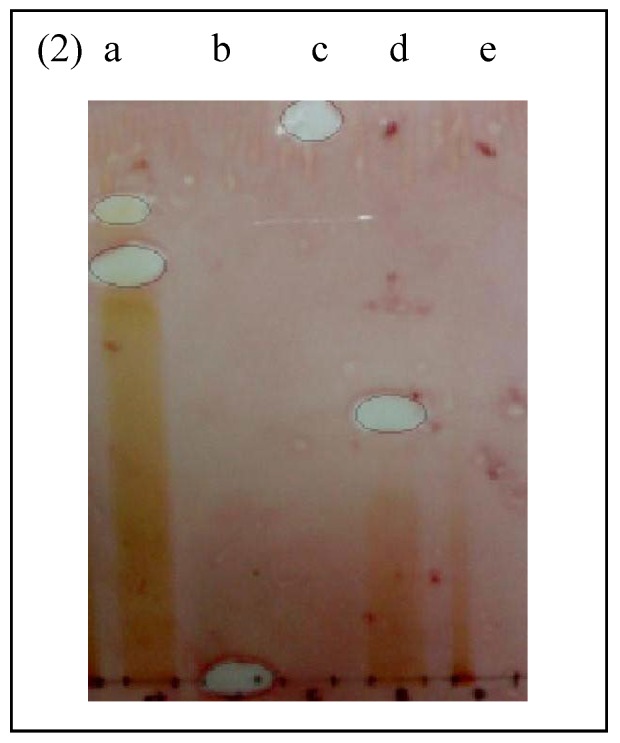
Bioautogram of fractions and antibiotics sprayed with actively growing *Escherichia coli.* **a**: ethyl acetate fraction. **b**: methicillin. **c**: vancomycin. **d**: butanol fraction. **e**: hexane fraction.

The standard antibiotics methicillin and vancomycin do not show any inhibition zones towards MRSA*.* This was in agreement with previous findings that proved vancomycin resistance developed in patients with MRSA infection [15]. In addition, all beta lactam antibiotics including methicillin are incapable of inhibiting MRSA [16]. Interestingly, the bioautogram against *E. coli* showed inhibition zones at Rf 0.77 (Figure 2) indicating the same compound was responsible for the antimicrobial activity against both Gram positive MRSA and Gram negative *E. coli*. *E. coli* was also inhibited by another compound (Rf 0.83) of the ethyl acetate fraction and butanol fraction (Rf 0.50) (Figure 2). The standard antibiotics methicillin and vancomycin showed inhibition zones against *E. coli*.

Subsequently an experiment was conducted to isolate, identify and determine the inhibitory concentration of the active compound that was capable of inhibiting both Gram positive MRSA and Gram negative *E. coli*. This compound was identified as xanthorrhizol (see Section 2.2) and its MIC value was found to be 25.0 μg/mL against MRSA and >200 µg/mL against *E. coli* (Table 3).

**Table 3:** MIC of CSLE, ethyl acetate fraction, xanthorrhizol and antibiotic against microorganisms.

M/o	CSLE (mg/mL)	Ethyl acetate fraction (μg/mL)	Xanthorrhizol (μg/mL)	Vancomycin	Methicillin
1	0.78	200	>200	12.5	12.5
2	0.78	100	12.5	12.5	12.5
3	0.78	100	25.0	>50	>50

M/o: Microorganisms. **1**: *Escherichia coli*. **2**: *Staphylococcus aureus*. **3**: methicillin resistant *Staphylococcus aureus.*

This value was in agreement with a previous study on the antimicrobial activity of xanthorrhizol [17]. The MIC value of xanthorrhizol against *E. coli* was higher than MIC of ethyl acetate fraction (200 μg/mL) on this bacterial sp. (Table 3). This might be due to some synergistic activity of the compound present in ethyl acetate fraction to inhibit *E. coli*.

### 2.2. Isolation and Identification of Antimicrobial Compound

**Figure 3:**
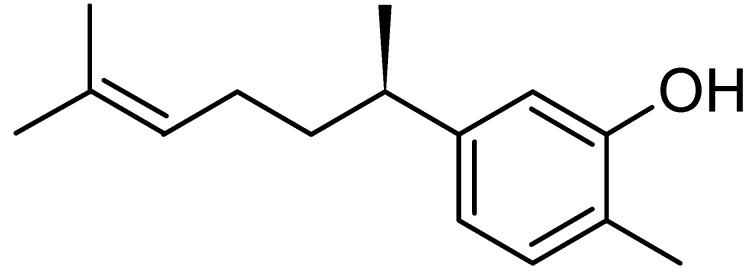
Structure of xanthorrhizol.

Phytochemical screening of the bioactive compound indicated that this compound was a terpenoid, since it showed brown colour after being sprayed with vanillin sulphuric reagent [18]. It was isolated by preparative TLC and identified by GC-MS, UV, FTIR and NMR as xanthorrhizol [phenol 5-(1,5-dimethyl-4-hexenyl)-2-methyl] (Figure 3). The obtained data was also in agreement with the spectroscopic results from other published articles on xanthorrhizol [19].

There are numerous reports on the presence of xanthorrhizol in the *Curcuma xanthorrhiza* rhizome [20,21,22,23]. The presence of xanthorrhizol in other plants such as *Iostephane heterophylla* root has also been reported [17]. Xanthorrhizol has been reported to possess antioxidant, anti-inflammatory, antibacterial, antimycotic and anticancer activity [20,21,22,23,24].

## 3. Experimental

### 3.1. General

Boiling point was determined on a micro-boiling point apparatus. Optical rotation was measured on a Perkin-Elmer 241 polarimeter. A UV spectrum was obtained on a UV-1800 series UV Shimadzu spectrophotometer. The functional group of bioactive compound was identified by Fourier Transform Infrared (FTIR) Spectroscopy (Nicolet 6700, USA) using potassium bromide (KBr). NMR spectra were recorded on a Varian Unity INOVA at 300 MHz (^1^H) or 75MHz (^13^C). Samples (2.0 mg/mL) were dissolved in deuterated methanol and spectrometric data recorded at 80 °C, 10,000 accumulations, pulse 15 μs, acquisition time 3 s and relaxation delay 5 s. Agilent Gas Chromatography (GC 6890N, China), and Agilent Mass Spectrometer (MS 5973I, USA) was equipped with capillary HP-5MS column, (30 m x 0.25 mm x 0.25 µm). The inlet temperature was set at 280 °C and Mass Selective Detector (MSD) transferline heater at 285 ºC. GC was performed in splitless mode. Flow rate of carrier gas (helium) was maintained at 1.2 mL/min. Initial temperature of oven was 70 °C and hold for 2 minute. Then it was increased to 280 °C by 20 °C/min and held for 20 min. Sample (1 µL) was injected and the fragmentation pattern of the compound peak was compare using the NIST02 library for identification.

### 3.2. Plant Materials

*C. iners* leaves were collected at Universiti Sains Malaysia (USM) in March 2009. The authentication was carried out by a botanist from School of Biological Sciences, USM where the plant material was deposited. The voucher specimen number is 11014.

### 3.3. Extraction and Fractionation Procedures

Powdered dried leaves (100 g) of the plant were macerated in methanol (500 mL) for 3 days. The leaves extract was filtered and concentrated under reduced pressure at 40 °C in a rotary evaporator. The concentrated extract obtained was placed in the oven for 3 days at 40 °C to remove the remaining methanol completely. The dried extract was standardized according to gas chromatography-mass spectrometry (GC-MS) method [11]. *Cinnamomum iners* standardized leaves extract (CSLE) was fractionated based on the polarity of the solvents as follows: extracted twice with 100 mL of *n*-hexane to obtain the hexane fraction. Then the same procedure was employed for ethyl acetate and butanol fractions.

### 3.4. Isolation and Characterization of Xanthorrhizol

The bioactive compound was isolated by preparative TLC using ethyl acetate fraction and the chromatogram was developed with ethyl acetate/methanol/water (10:1.35:1) (v/v) solvent system and air-drying. Antibacterial compound that shows inhibition zones was scrapped out and dissolved in methanol prior to the centrifuge. The supernatant collected was evaporated in the oven followed by freeze drying. The isolated compound was identified by GC-MS, UV, FTIR and NMR The pure compound obtained as pale yellow colour oil, boiling point 327 °C. The specific rotation was (*R*)-(-)-Xanthorrhizol: [α]^24^_D_ -53.7° (c = 0.80, CHC1_3_). The UV spectrum in CHC1_3_ showed absorptions at γ_max_ 276 nm, 217.5 nm and 245.5 nm which indicate a phenolic group. The IR spectrum of this compound showed characteristic absorption bands at 3,382.8 cm^-1 ^which indicate an alcoholic function. The spectrum also had representative bands at 1604.9, 1503.0 and 1375.2 cm^-1^ The ^1^H-NMR spectra of the compound gave following values: δ 1.21 (3H, d, *J* = 6.9 Hz), 1.56-1.67 (2H, m), 1.70 (6H, s), 1.75-1.92 (2H, m), 2.24 (3H, s), 2.58-2.69 (1H, m, *J* = 7.0 Hz), 5.08-5.14 (1H, t), 6.63 (1H, s), 6.70 (1H, dd, *J* = 1.3, 7.7 Hz), 7.04 (1H, d, *J* = 7.6 Hz). ^13^C-NMR: δ 15.34, 17.68, 22.38, 25.71, 26.15, 38.38, 39.03, 113.54, 119.41, 120.85, 124.52, 130.76, 131.42, 147.21, 153.64. Xanthorrhizol was detected at 9.52 min retention time in GC. Mass spectroscopy showed the major characteristic fragmentations (m/z = 41; m/z = 55; m/z = 91; m/z = 121; m/z = 136; m/z = 148; m/z = 161; M^+^ = 218) pattern which is exactly identical to xanthorrhizol [phenol 5-(1,5-dimethyl-4-hexenyl)-2-methyl] mass fragmentation pattern with molecular weight C_15_H_22_O. When pure xanthorrhizol (purchased from Sigma) was injected in GC, the peak appeared exactly at same retention time (9.52 min) as bioactive compound.

### 3.5. Microbial Strains

The microorganisms used in this study included *Bacillus cereus* (ATCC 10876), *Bacillus subtilis*, *Pseudomonas aeruginosa* (ATCC 27853), *Escherichia coli* (ATCC 25922), *Staphylococcus aureus* (ATCC 12600), *Shigella sonnei*, *Candida albicans* and *Saccharomyces cerevisiae* were obtained from Microbiology lab, School of Biology, Universiti Sains Malaysia. The methicillin resistant *Staphylococcus aureus* (MRSA) and *Salmonella typhi* strains used in this study were clinical isolates from Penang General Hospital (Penang, Malaysia). Bacterial cultures were maintained on Mueller-Hinton agar (MHA) and yeast cultures were maintained in sabouraud dextrose agar (SDA) at 4 °C. Sub-culturing was done weekly. The cells were inoculated in MH broth for bacteria (37 °C, 18 h) or SD broth for yeast (35 °C, 48 h) prior to the screening procedure.

### 3.6. Disk Diffusion Assay

The experiment was performed according to the method reported of by Alzoreky *et al*. [25]. For the determination of antibacterial and antifungal activity, cultures were adjusted to10^6^ colony-forming units (CFU)/mL of bacteria or 2 × 10^5^ CFU/mL of yeast cell using 0.5 McFarland standards. Subsequently, cultures were inoculated into MHA for bacteria or SDA for yeast by spreading. Then sterile disks were impregnated with CSLE, fractions (50 mg/mL, 25 µL) or standards. Chloramphenicol (for bacteria) and miconazole nitrate (for yeast) at 30 µg/mL were used as standard antibiotics. Disc impregnated with methanol alone in the center served as a control. All the plates were incubated at 37 ºC for 24 h for bacteria and 35 °C for 48 h for yeast. The diameter of inhibitory zones (including the diameter of the discs) on the agar surface around the discs were measured after the incubation period and values >7 mm were considered as active against microorganisms. All of the experiments were performed in triplicate.

### 3.7. Determination of Minimum Inhibitory Concentrations (MIC)

For the determination of minimum inhibitory concentration (MIC), the broth dilution method as described by Eloof [26] was employed. CSLE, fractions, isolated compound and antibiotics standard were serially diluted in microplates wells to acquire concentrations of CSLE (0.78-50.00 mg/mL), fractions (100-800 µg/mL), isolated compound (6.25-200.00 µg/mL) and antibiotics standards (3.13-50 µg/mL). Cultures were adjusted to achieve an inoculums size of 10^6 ^colony forming unit per mL (CFU/mL) for bacteria or 2 × 10^5^ CFU/mL yeast cell using 0.5 McFarland standards. 5 µL of culture was added to each well. Then, the microplates were placed for 24 h incubation at 37 °C for bacteria or 48 h at 35 °C for yeast. After the overnight incubation, *p*-iodonitrotetrazolium violet (INT) (0.5 mg/mL, 20 µL) was added to the microplate wells. The formation of red chromogen indicates the occurrence of microorganisms. The MIC value was determined as the lowest concentration of the test sample in the well that did not form red colour.

### 3.8. Determination of Minimum Bactericidal and Fungicidal Concentration (MBC and MFC)

The minimum bactericidal and fungicidal concentration of CSLE was determined according to the method of Doughari [27]. For the determination of MBC and MFC, a loopful of broth was collected from the well with no visible growth of bacteria or yeast and inoculated to sterile MHA (for bacteria) and SDA (for yeast) by spreading using swab sticks. After the overnight incubation, the concentration at which no visible growth or fewer than three colonies observed was noted as the minimum bactericidal concentration (MBC) for bacteria or minimum fungicidal concentration (MFC) for yeast.

### 3.9. Bioautography and Identification of Antimicrobial Compound

This test was performed only on selected bacterial cultures that were remarkably inhibited by CSLE and fractions according to the method of Ishikawa [28]. CSLE and fractions (50 mg/mL) were applied over a chromatosheet (Merck pre coated silica gel 60 plates, F254; layer thickness 0.20 mm for analytical TLC) followed by elution with a system of ethyl acetate/methanol/water (10:1.35:1) and air-drying. The plates were run in duplicate, one set was used as the reference chromatogram and the other was used for bioautography. The spots in the chromatogram were visualized in the UV chamber (wavelength 365 and 254 nm) and using vanillin sulphuric reagent. The chromatogram was sprayed with suspension of MRSA and *E. coli* bacteria that was adjusted to an inoculums size of 10^6^ CFU/mL using 0.5 McFarland standards. The plate was incubated at 37 °C for 24 h. The inhibition zones were visualized by spraying with *p*-iodonitrotetrazolium violet (INT, 2.0 mg/mL) and the Rf values of the bioactive compounds were determined.

## 4. Conclusions

The findings of this study have provided an insight into the antibacterial properties of *Cinnamomum iners* leaves extracts, especially for MRSA strains. It is also worth noting that this is the first report on the presence of xanthorrhizol in the *Cinnamomum iners* leaves.

## References

[1] Buzby J.C., Roberts, T. (1997). Economic costs and trade impacts of microbial foodborne illness. World Health Stat. Q..

[2] Levy S.B. (1998). The challenge of antibiotic resistance. Sci. Am..

[3] Cowan M.M. (1999). Plant products as antimicrobial agents. Clin. Microbiol. Rev..

[4] Holah J.T., Taylor J.H., Dawson D.J., Hall K.E. (2002). Biocide use in the food industry and the disinfectant resistance of persistent strains of *Listeria monocytogenes* and *Escherichia coli*. J. Appl. Microbiol..

[5] Styers D., Sheehan D.J., Hogan P., Sahm D.F. (2006). Laboratory-based surveillance of current antimicrobial resistance patterns and trends among *Staphylococcus aureus*: 2005 status in the United States. Ann. Clin. Microbiol. Antimicrob..

[6] Deresinski S. (2005). Methicillin-resistant *Staphylococcus aureus*: An evolutionary, epidemiologic, and therapeutic odyssey. Clin. Infect. Dis..

[7] Choi O.H. (2003). Tumbuhan liar, khasiat ubatan dan kegunaan lain.

[8] Wan O.A., Ngah Z.U., Zaridah M.Z., Noor R.A. (2007). *In vitro* and *in vivo* antiplasmodial properties of some Malaysian plants used in traditional medicine. Infect. Dis. J. Pak..

[9] Iida N., Ishii R., Hakamata J., Myamoto S., Oozeki H. (1997). Amylase inhibitors for food and pharmaceutical. Japanese Kokai Tokkyo Koho.

[10] Baruah A., Nath S.C., Hazarika A.K. (2001). Stem bark oil of *Cinnamomum iners* Reinw. Indian Perfumer..

[11] Mustaffa F., Indurkar J., Ismail S., Mordi M.N., Surash R., Mansor S.M. (2010). Analgesic activity, toxicity study and phytochemical screening of *Cinnamomum iners* standardized leaves methanolic extract. Pharmacogn. Res..

[12] Mustaffa F., Indurkar J., Ismail S., Mordi M.N., Surash R., Mansor S.M. (2010). Antioxidant capacity and toxicity screening of *Cinnamomum iners* standardized leaves methanolic extract. Int. J. Pharmacol..

[13] Pengelly A. (2004). Constituents of Medicinal Plants.

[14] Eloff J.N., Masoko P. (2005). The diversity of antifungal compounds of six South African Terminalia species (Combretaceae) determined by bioautography. Afr. J. Biotechnol..

[15] Sieradzki K., Roberts R.B., Haber S.W., Tomasz A. (1999). The development of vancomycin resistance in a patient with methicillin-resistant *Staphylococcus aureus* infection. N. Engl. J. Med..

[16] Shanab B.A., Adwan G., Jarrar N., Hijleh A.A., Adwan K. (2006). Antibacterial activity of four plant extracts used in Palestine in folkloric medicine against methicillin-resistant *Staphylococcus aureus*. Turk. J. Biol..

[17] Mata R., Martinez E., Bye R., Morales G., Singh M.P., Janso J.E., Maiese W.M., Timmermann B. (2001). Biological and mechanistic activities of xanthorrhizol and 4-(1,5-dimethylhex-4-enyl)-2-methylphenol isolated from *Iostephane Heterophylla*. J. Nat. Prod..

[18] Wagner H., Bladt S. (2001). Plant Drug Analysis: A Thin Layer Chromatography Atlas.

[19] Ehara T., Tanikawa S., Ono M., Akita H. (2007). Synthesis of (R)-curcumene and (R)-xanthorrhizol based on 1, 2-aryl migration via phenonium ion. Chem. Pharm. Bull..

[20] Hwang J.K., Shim J.S., Pyun Y.R. (2000). Antibacterial activity of xanthorrhizol from *Curcuma xanthorriza* against oral pathogens. Fitoterapia.

[21] Rukayadi Y., Hwang J.K. (2007). *In vitro* antimycotic activity of xanthorrhizol isolated from *Curcuma xanthorriza* Roxb. against opportunistic filamentous fungi. Phytother. Res..

[22] Lim C.S., Jin D.Q., Mok H., Oh S.J., Lee J.U., Hwang J.K., Ha I., Han J.S. (2005). Antioxidant and antiinflammatory activities of xanthorrhizol in Hippocampal neurons and primary cultured microglia. J. Neurosci. Res..

[23] Rukayandi Y., Yong D., Hwang J.K. (2006). *In vitro* anticandidal activity of xanthorrhizol isolated from *Curcuma xanthorrhiza* Roxb. J. Antimicrob. Chemother..

[24] Oh H.I., Shim J.S., Gwon H.S., Kwon H.J., Hwang J.K. (2009). The effect of xanthorrhizol on the expression of matrix metalloproteinase-1 and type-I procollagen in ultraviolet-irradiated human skin fibroblasts. Phytother. Res..

[25] Alzoreky N.S., Nakahara K. (2003). Antibacterial activity of extracts from some edible plant commonly consumed in Asia. Int. J. Food Microbiol..

[26] Eloff J.N. (1998). A sensitive and quick microplate method to determine the minimal inhibitory concentration of plant extracts for bacteria. Planta Med..

[27] Doughari J.H. (2006). Antimicrobial activity of *Tamarindus indica* Linn. Trop. J. Pharm. Res..

[28] Ishikawa N.K., Kasuya M.C.M., Vanetti M.C.D. (2001). Antibacterial activity of *Lentinula edodes* grown in liquid medium. Braz. J. Microbiol..

